# Prolonged Delayed Graft Function Is Associated with Inferior Patient and Kidney Allograft Survivals

**DOI:** 10.1371/journal.pone.0144188

**Published:** 2015-12-17

**Authors:** Tainá Veras de Sandes-Freitas, Cláudia Rosso Felipe, Wilson Ferreira Aguiar, Marina Pontello Cristelli, Hélio Tedesco-Silva, José Osmar Medina-Pestana

**Affiliations:** 1 Nephrology Division, Hospital do Rim / Universidade Federal de São Paulo–UNIFESP, São Paulo, Brazil; 2 Urology Division, Hospital do Rim / Universidade Federal de São Paulo–UNIFESP, São Paulo, Brazil; University of Toledo, UNITED STATES

## Abstract

It is unclear if there is an association between the duration of delayed graft function (DGF) and kidney transplant (KT) outcomes. This study investigated the impact of prolonged DGF on patient and graft survivals, and renal function one year after KT. This single center retrospective analysis included all deceased donor KT performed between Jan/1998 and Dec/2008 (n = 1412). Patients were grouped in quartiles according to duration of DGF (1–5, 6–10, 11–15, and >15 days, designated as prolonged DGF). The overall incidence of DGF was 54.2%. Prolonged DGF was associated with retransplantation (OR 2.110, CI95% 1.064–4.184,p = 0.033) and more than 3 HLA mismatches (OR 1.819, CI95% 1.117–2.962,p = 0.016). The incidence of acute rejection was higher in patients with DGF compared with those without DGF (36.2% vs. 12.2%, p<0.001). Compared to patients without DGF, DGF(1–5), DGF(6–10), and DGF(11–15), patients with prolonged DGF showed inferior one year patient survival (95.2% vs. 95.4% vs. 95.5% vs. 93.4% vs. 88.86%, p = 0.003), graft survival (91% vs. 91.4% vs. 92% vs. 88.7% vs. 70.5%, p<0.001), death-censored graft survival (95.7% vs. 95.4% vs. 96.4% vs. 94% vs. 79.3%, p<0.001), and creatinine clearance (58.0±24.6 vs. 55.8±22.2 vs. 53.8±24.1 vs. 53.0±27.2 vs. 36.8±27.0 mL/min, p<0.001), respectively. Multivariable analysis showed that prolonged DGF was an independent risk factor for graft loss (OR 3.876, CI95% 2.270–6.618, p<0.001), death censored graft loss (OR 4.103, CI95% 2.055–8.193, p<0.001), and death (OR 3.065, CI95% 1.536–6.117, p = 0.001). Prolonged DGF, determined by retransplantation and higher HLA mismatches, was associated with inferior renal function, and patient and graft survivals at one year.

## Introduction

Several studies have evaluated the impact of delayed graft function (DGF) on long-term clinical outcomes. While the effect of DGF on patient survival remains unclear, there is a consensus regarding its association with inferior graft survival. In a recent meta-analysis, patients who developed DGF had a 41% increased risk of graft loss at a mean follow up time of 3.2 years [1]. Notably, a recent study proposed a causal association between DGF and graft failure [2]. According to this hypothesis, ischemia-reperfusion injury may cause increased MHC class I and II expression, increasing the risk of acute rejection (AR) [3,4]. Persistent activation of the host immune response has been associated with immune mediated interstitial fibrosis and tubular atrophy (IF/TA). Finally, maladaptive repair of parenchymal and tubular cells after acute kidney injury may also contribute to fibrosis and permanent loss of functioning renal mass, increasing the risk of late graft failure [5].

Initial allograft function is better explained as a continuous variable, ranging from immediate graft function observed after living donation to prolonged periods of dialysis-dependency seen after transplantation of kidneys recovered from expanded criteria donor and prolonged cold ischemia times. Therefore, the severity of clinical presentation represents the spectrum of the same disease, which is directly associated with prognosis. This hypothesis is supported by the inferior outcomes observed in patients with DGF compared with to those with slow graft function and immediate renal function [6]. Intuitively, the time elapsed to recover renal function may reflect the severity of the injury and may be associated with outcome. Nevertheless, studies investigating the duration of DGF and outcomes have used different criteria for time on DGF, have not analyze risk factors for the development of prolonged DGF and have also shown conflicting results [7–10].

In a recent retrospective cohort analysis of 1518 recipients of kidneys recovered from brain-dead donors we reported a high incidence of DGF of 57.3%, which was primarily associated with inadequate donor maintenance before organ donation [11]. The aim of this analysis was to determine the risk factors associated with the incidence and duration of DGF and their association with kidney transplants function and outcomes one year after transplantation.

## Methods

### Study design

This study analyzed data from all recipients of deceased donor kidney transplants performed between January 1^st^ 1998 and December 31^st^ 2008 at Hospital do Rim, São Paulo, Brazil, and was approved by the local Ethics Committee (Comitê de Ética em Pesquisa, Universidade Federal de São Paulo). Data were retrospectively collected by systematic review of medical charts and electronic database. Due to the retrospective nature of the study, the informed consent was not obtained. Patient records and information was anonymized and de-identified prior to analysis. None of the transplant donors were from a vulnerable population and all donors or next of kin provided written informed consent that was freely given. Of 1736 deceased donor kidney transplants performed in this period, 324 were excluded due to incomplete information. All deceased donors were brain dead (DBD) and the recovered kidneys were preserved in static cold storage solution.

### Objectives

The main objective was the analysis of 1-year graft and patient survivals according to the duration of DGF period. Secondary objectives included risk factors for DGF and prolonged DGF, incidence of AR and renal function.

### Definitions

Delayed graft function was defined as the requirement of at least one dialysis session during the first week after transplantation, regardless of the clinical indication [12]. DGF duration was computed up from the day of transplantation to the last dialysis section. Patients were grouped in quartiles according to the time on DGF as follows: patients without DGF (without DGF), patients with DGF duration up to 5 days (DGF 1–5 days), between 6 and 10 days (DGF 6–10 days); between 11 and 15 days (DGF 11–15 days), and longer than 15 days, here denominated prolonged DGF. In patients with DGF, surveillance biopsies were performed routinely until graft function recovery. All treated AR episodes were included in the analysis, including those confirmed or not by histopathological evaluation. Renal function was determined by creatinine clearance calculated using the Cochroft-Gault formula.

### Statistical Analysis

Continuous variables were presented as mean and standard deviation and compared using ANOVA, with Tukey *post hoc* test. Categorical variables were presented as frequency and percentage and compared using Chi-square or Fisher tests. Survival curves were obtained using Kaplan-Meier method and compared using the log rank test. The comparison of creatinine clearance values over time between groups was performed using analysis of variance for repeated measures. The last observation carried forward (LOCF) analysis was used for missing estimated glomerular filtration rate (eGFR) values, attributing zero to patients who lost the graft and the last available value for those who died or lost follow up. A multivariable logistic regression model was fitted to compute covariate-adjusted odds ratios (OR) for the following outcomes: DGF, prolonged DGF, patient death, graft loss, and death-censored graft loss.

Fifteen variables were included in the model for DGF and prolonged DGF risk analysis: recipient age, history of diabetes, time on dialysis, panel reactive antibodies (PRA), re-transplantation, human leukocyte antigen (HLA) mismatches, cold ischemia time, anti-thymocyte globulin (ATG) induction therapy, and calcineurin inhibitor (CNI)-based immunosuppressive regimen; donor age, cerebrovascular death, final serum creatinine, history of hypertension, use of vasoactive drugs, and cardiac arrest prior to recovery.

Seventeen variables were included in the model for patient death, graft loss, and death-censored graft loss risk analysis as follows: recipient age, recipient gender, recipient history of diabetes, time on dialysis, PRA, re-transplantation, donor age, cerebrovascular death, final serum creatinine, donor history of hypertension, cardiac arrest prior to retrieval, ATG induction therapy, CNI-based immunosuppressive regimen, mycophenolate use, cold ischemia time, AR, and time on DGF.

A p-value of <0.2 in univariable analysis was considered statistically significant for including variables in multivariable analysis. For all other analysis, a p-value of <0.05 was considered statistically significant. Statistical analysis was performed using SPSS v.18.0 software (SPSS, Inc., Chicago, IL, USA).

## Results

### Demographic characteristics

Of the 1412 patients included, 765 (54.2%) presented DGF. The recipients in the DGF group showed higher mean age, higher prevalence of African-American ethnicity, chronic kidney disease attributed to hypertension or diabetes and re-transplantation, and higher mean time on dialysis. Donors in DGF group showed higher mean age, higher prevalence of hypertension and cerebrovascular death and higher mean final serum creatinine. Recipients in the DGF group were transplanted with longer cold ischemia time, a higher proportion received ATG induction therapy but fewer patients received initial immunosuppressive regimen based on CNI (Table 1).

**Table 1:** Demographic and clinical characteristics.

	Without DGF	DGF	DGF 1–5 days	DGF 6–10 days	DGF 11–15 days	DGF >15 days
	N = 647	N = 765	N = 197	N = 224	N = 151	N = 193
**Gender–male, n(%)**	343 (53)	436 (57)	124 (62.9)	123 (54.6)	87 (57.6)	102 (52.8)
**Age (years, mean±SD)**	38.5±17.7	43±14.7^#^	44.3±14.9	43.5±14	41.4±15.5	42.2±14.5
**Ethnicity, n(%)**						
*Caucasian*	347 (53.6)	371 (48.5)*	103 (52.3)	120 (53.6)	80 (53)	68 (35.2)^§^
*Afro-American*	163 (25.2)	173 (22.6)	40 (20.3)	47 (21)	34 (22.5)	52 (26.9)
*Mixed*	50 (7.7)	90 (11.8)	28 (14.2)	21 (9.4)	17 (11.3)	24 (12.4)
*Asian*	11 (1.7)	9 (1.2)	1 (0.5)	1 (0.4)	2 (1.3)	5 (2.6)
*No available*	76 (11.7)	122 (15.9)	25 (12.7)	35 (15.6)	18 (11.9)	44 (22.8)
**ESRD Etiology, n(%)**						
*Unknown*	242 (37.4)	248 (32.4)*	70 (35.5)	72 (32.1)	50 (33.1)	56 (29)
*Hypertension*	82 (12.7)	132 (17.3)	37 (18.8)	35 (15.6)	23(15.2)	37 (19.2)
*Diabetes*	63 (9.7)	104 (13.6)	25 (12.7)	32 (14.3)	21 (13.9)	26 (13.5)
*Glomerulonephritis*	138 (21.3)	156 (20.4)	35 (17.8)	45 (20.1)	30 (19.9)	46 (23.8)
*PKD*	42 (6.5)	45 (5.9)	13 (6.6)	16 (7.1)	6 (4)	10 (5.2)
*Urological*	46 (7.1)	55 (7.2)	10 (5.1)	17 (7.6)	17 (11.3)	11 (5.7)
*Displasia*	17 (2.6)	9 (1.2)	4 (2)	2 (0.9)	2 (1.3)	1 (0.5)
*Other*	17 (2.6)	16 (2.1)	3 (1.5)	5 (2.2)	2 (1.3)	6 (3.1)
**Time on dialysis (months, mean±SD)**	49.5±37.1	64±41.6^#^	59.7±36.2	58.7±35.4	61.5±39.4	76.4±51.5^¶^
**Re-transplantation, n(%)**	34 (5.3)	76 (9.9)*	14 (7.1)	13 (5.8)	12 (7.9)	37 (19.2)^¶^
**PRA class I (mean±SD)**	7.7±18.8	9.3±20.9	10.1±22.8	7.5±18.6	8.3±19.5	11.5±22.3
**PRA class II (mean±SD)**	3.1±14.9	4.7±17.8	6.4±22.6	3.3±14.5	5.6±19.6	3.7±14
**HLA Mismatches**	2.7±1.4	2.7±1.4	2.6±1.3	2.6±1.3	2.8±1.3	3±1.5^§^
**Donor age (years, mean±SD)**	33.4±18	39.7±15.4^#^	42.7±15.3	39.1±15.7	38.3±15.1	38.6±15^§^
**Donor death cause, n(%)**						
*Cerebrovascular*	283 (43.7)	410 (53.6)^#^	118 (59.9)	125 (55.8)	72 (47.7)	95 (49.2)
*Traumatic*	293 (45.3)	314 (41)	71 (36)	86 (38.4)	72 (47.7)	85 (44)
*Anoxic encephalopathy*	28 (4.3)	16 (2.1)	3 (1.5)	5 (2.2)	4 (2.6)	4 (2.1)
*Neoplasia*	5 (0.8)	3 (0.4)	1 (0.5)	1 (0.4)	1 (0.7)	0 (0)
*Meningitis*	13 (2)	11 (1.4)	2 (1)	3 (1.3)	0 (0)	6 (3.1)
*Other*	25 (3.9)	11 (1.4)	2 (1)	4 (1.8)	2 (1.3)	3 (1.6)
**Donor—Hypertension, n(%)**	115 (17.8)	214 (28)^#^	70 (35.5)	58 (25.9)	40 (26.5)	46 (23.8)^§^
**Final sCR (mg/dL,mean ± SD)**	1.2±0.8	1.6±1.1^#^	1.6±1.1	1.6±1.1	1.7±1.5	1.4±0.8
**Final sCR > 1.5 mg/dL, n(%)**	148 (22.9)	294 (38.4)^#^	80 (40.6)	87 (38.8)	63 (41.7)	64 (33.2)
**Expanded criteria donor, n(%)**	93 (14.4)	140 (18.3)	51 (25.9)	38 (17)	22 (14.6)	29 (15)^§^
**Vasoactive drug use, n(%)**	575 (88.9)	167 (84.8)	167 (84.8)	208 (92.9)	136 (90.1)	163 (84.5)^§^
**Cardiac arrest pior to recovery, n(%)**	109 (16.8)	107 (14)	23 (11.7)	33 (14.7)	27 (17.9)	24 (12.4)
**DCD, n (%)**	0 (0)	0 (0)	0 (0)	0 (0)	0 (0)	0 (0)
**CIT (hour, mean±SD)**	22±6.5	24.3±7.1^#^	24.1±6.9	25.1±6.8	24±7.2	22.9±7.5^§^
**Time on DGF (days, mean±SD)**	0 (0)	13.2±13.8	2.7±1.4	8.1±1.4	12.9±1.3	30±18^¶^
**ATG induction, n(%)**	70 (10.8)	155 (20.3)^#^	51 (25.9)	39 (17.4)	26 (17.2)	39 (20.2)
**CNI-based regimen, n(%)**	579 (89.5)	592 (77.4)^#^	140 (71.1)	168 (75)	124 (82.1)	160 (82.9)^§^
**Initial ISS, n (%)**						
*CNI-ST-AZA*	338 (52.2)	291 (38.0) ^#^	68 (34.5)	74 (33.0)	61 (40.4)	88 (45.6) ^§^
*CNI-ST-MPA*	220 (34.0)	271 (35.4)	65 (33.0)	86 (38.4)	58 (38.4)	62 (32.1)
*ST-MPA*	52 (8.0)	148 (19.3)	55 (27.9)	49 (21.9)	22 (14.6)	22 (11.4)
*CNI-ST-SRL*	12 (1.9)	16 (2.1)	1 (0.5)	5 (2.2)	3 (2.0)	7 (3.6)
*Other*	25 (3.9)	39 (5.1)	8 (4.1)	10 (4.5)	7 (4.6)	14 (7.3)
**ST-sparing regimens, n(%)**	4 (0.6)	5 (0.6)	2 (1.0)	3 (1.3)	0 (0)	0 (0)

ATG: anti-thymocyte globulin; AZA: azathioprine; CIT: cold ischemia time; CNI: calcineurin inhibitor; DCD: donation after cardiac death; DGF: delayed graft function; ESRD: end stage renal disease; HLA: human leukocyte antigen; ISS: immunosuppressive regimen; MPA: mycophenolate mofetil or sodium; PKD: polycystic kidney disease; PRA: panel reactive antibodies; sCR: serum creatinine; SD: standard deviation; SRL: sirolimus; ST: steroid.

*p<0.05 vs. without DGF group.

^#^ p<0.001 vs. without DGF group.

^§^ p<0.005 among all DGF groups.

^¶^ p<0.001 among all DGF groups.

Of the 765 patients with DGF, 197 (25.8%) were in DGF 1–5 days, 224 (29.3%) in DGF 6–10 days, 151 (19.7%) in DGF 11–15 days, and 193 (25.2%) in DGF > 15 days groups. The mean time on DGF in these groups was 2.7±1.4, 8.1±1.4, 12.9±1.3, and 30±18 days, respectively. Patients in the DGF>15 days group showed higher mean time on dialysis (76.4±51.5 months) when compared to DGF 1–5 days (59.7±36.2 months, p<0.001), DGF 6–10 days (58.7±35.4 months, p<0.001) and DGF 11–15 days groups (61.5±39.4 months, p = 0.005). Patients in DGF>15 days group also presented inferior percentage of Caucasian (35.2% vs. 52.3%, p = 0.002; vs. 53.6%, p = 0.003; vs. 53%, p = 0.010, respectively) and higher proportion of retransplantation when compared with other DGF groups (19.2% vs. 7.1%, p<0.001; vs. 5.8%, p<0.001; vs. 7.9%, p = 0.003, respectively) and higher HLA mismatches when compared to DGF 6–10 days group (3.0±1.5 vs. 2.6±1.3, p = 0.044). Compared to DGF 1–5 days group, donors in DGF>15 days group were younger (38.6±15 vs. 42.7±15.3, p = 0.042), and less frequently had hypertension (23.8% vs. 35.5%, p = 0.015) and were expanded criteria donors (15% vs. 25.9%, p = 0.009). Compared with DGF 6–10 days, a lower proportion of donors in DGF >15 days group received vasoactive drugs (84.5% vs. 92.9%, p = 0.018) and the cold ischemia time was shorter (22.9±7.5 vs. 25.1±6.8 hours, p = 0.012). A higher percentage of patients in DGF >15 days group received CNI-based regimen when compared with DGF 1–5 days group (82.9% vs. 71.1%, p = 0.006) (Table 1).

### Risk factors associated with DGF and prolonged DGF

Multivariable analysis showed that retransplantation, donor age, final serum creatinine, cold ischemia time, and CNI-based immunosuppressive regimen were the variables independently associated with DGF. Only retransplantation and HLA mismatches were associated with prolonged DGF (Table 2).

**Table 2:** Multivariable analysis for risk evaluation of DGF and prolonged DGF.

	DGF		Prolonged DGF	
	Univariable	Multivariable	Univariable	Multivariable
	p	OR (95% CI), p	p	OR (95% CI), p
**Age (years)**	<0.001	ns	0.413	
**Diabetes (yes/no)**	0.095	ns	0.954	
**Time on dialysis (months)**	<0.001	ns	<0.001	ns
**PRA (%)**	0.092	ns	0.341	
**Retransplantation (yes/no)**	0.001	2.132 (1.178–3.857), 0.012	<0.001	2.110 (1.064–4.184), 0.033
**HLA Mismatches >3 (yes/no)**	0.058	ns	0.014	1.819 (1.117–2.962), 0.016
**Donor age (years)**	<0.001	1.015 (1.006–1.024), 0.001	0.231	
**Cerebrovascular donor death (yes/no)**	<0.001	ns	0.159	ns
**Final sCR (mg/dL)**	<0.001	1.541 (1.305–1.820), <0.001	0.017	ns
**Donor–hypertension (yes/no)**	<0.001	ns	0.139	ns
**Vasoactive drugs use (yes/no)**	0.417		0.018	ns
**Cardiac arrest prior to recovery (yes/no)**	0.137	ns	0.473	
**Cold ischemia time (hours)**	<0.001	1.047 (1.024–1.070), <0.001	0.012	ns
**ATG induction (yes/no)**	<0.001	ns	0.983	
**CNI-based regimen (yes/no)**	<0.001	0.495 (0.341–0.719), <0.001	0.035	ns

ATG: anti-thymocyte globulin; DGF: delayed graft function; HLA: human leukocyte antigen; PRA: panel reactive antibodies; sCR: serum creatinine; CNI: calcineurin inhibitor; ns: not significant.

### Kidney transplant outcomes

**Fig 1:**
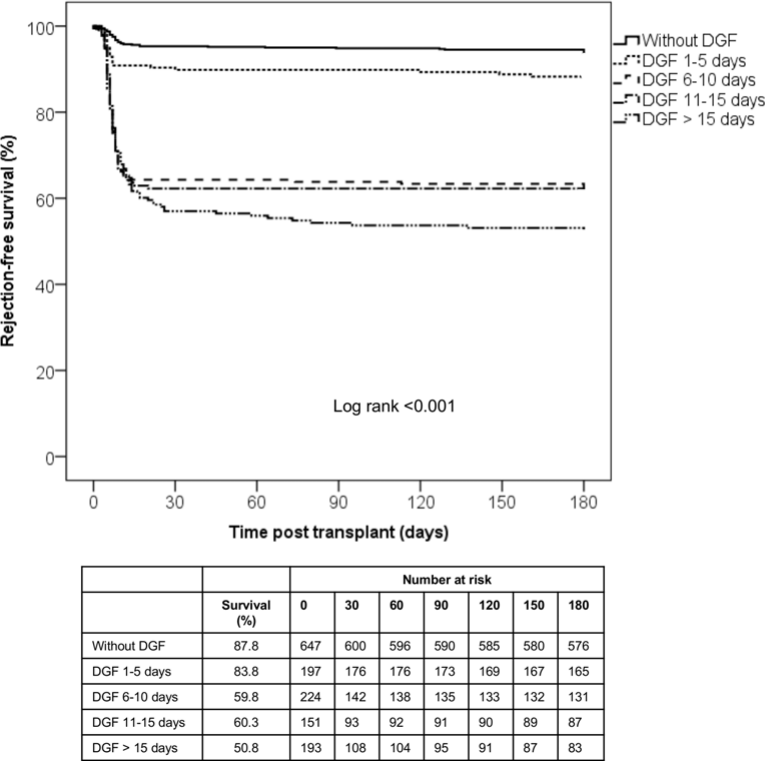
6-months rejection-free survival according to time on DGF. Patients with DGF longer than 15 days presented inferior rejection-free survival comparing with patients without DGF or DGF up to 5 days. Most episodes occurred within 30 days after transplant.

The incidence of acute rejection at 6 months was 3 times higher in patients with DGF compared with patients without DGF (36.2% vs. 12.2%, p<0.001). Among DGF groups, patients in DGF 1–5 days group presented lower incidence of acute rejection (16.1%) when compared to DGF 6–10 days (40.2%, p<0.001), DGF 11–15 days (39.7%, p<0.001) and DGF>15 days group (49.2%, p<0.001). Similarly, significant differences were observed in rejection free survival. Inspection of the survival curves revealed that the majority of acute rejection episodes occurred during the DGF period (Fig 1).

**Fig 2:**
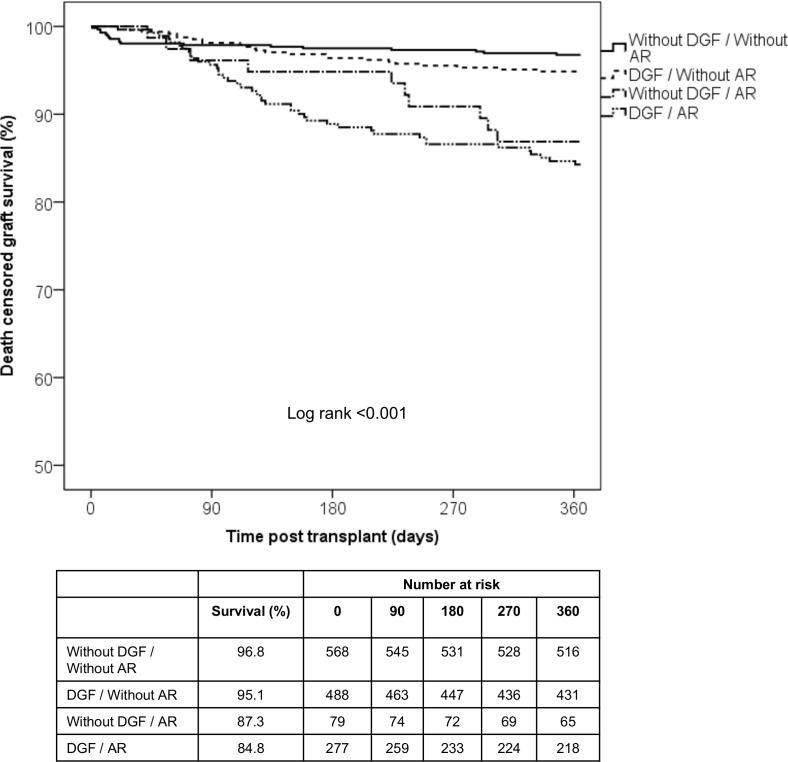
1-year death censored graft survival according to DGF and AR episodes. Patients with AR episodes and those with DFG and AR episodes presented inferior death censored graft survival.

Patients who presented acute rejection showed inferior death-censored graft survival (88%) compared with patients without acute rejection (95.8%, log rank p<0.001). Overall graft survival was also inferior (77.8% vs. 84.3%, log rank p = 0.005) but no difference was observed in patient survival (93.2% vs. 91.8%, log rank p = 0.366). Likewise, patients with DGF had inferior 1-year death-censored graft survival (91.4% vs. 95.7%, log rank p = 0.001). Overall graft survival was also inferior (85.8% vs. 91%, p = 0.003) but no difference was observed in patient survival (93.3% vs. 95.2%, p = 0.124). When we combined these two variables, only patients with acute rejection and those with both acute rejection and DGF showed inferior 1-year death-censored graft survival (96.8% vs. 95.1% vs. 87.3% vs. 84.5%, respectively, log rank p<0.001), respectively (Fig 2).

**Fig 3:**
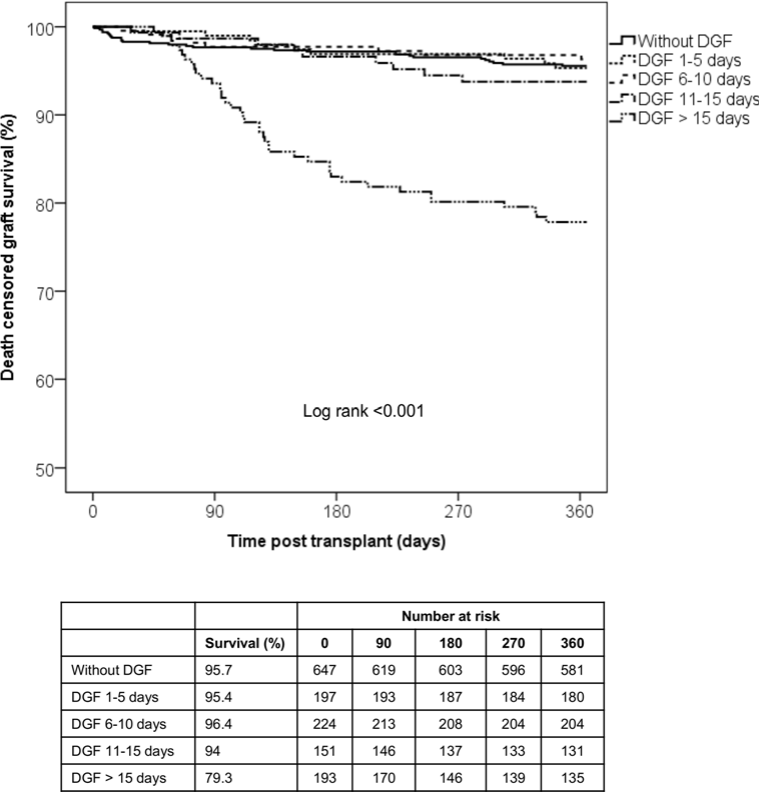
1-year death censored graft survival according to time on DGF. Only patients with DGF longer than 15 days presented inferior death censored graft survival.

**Fig 4:**
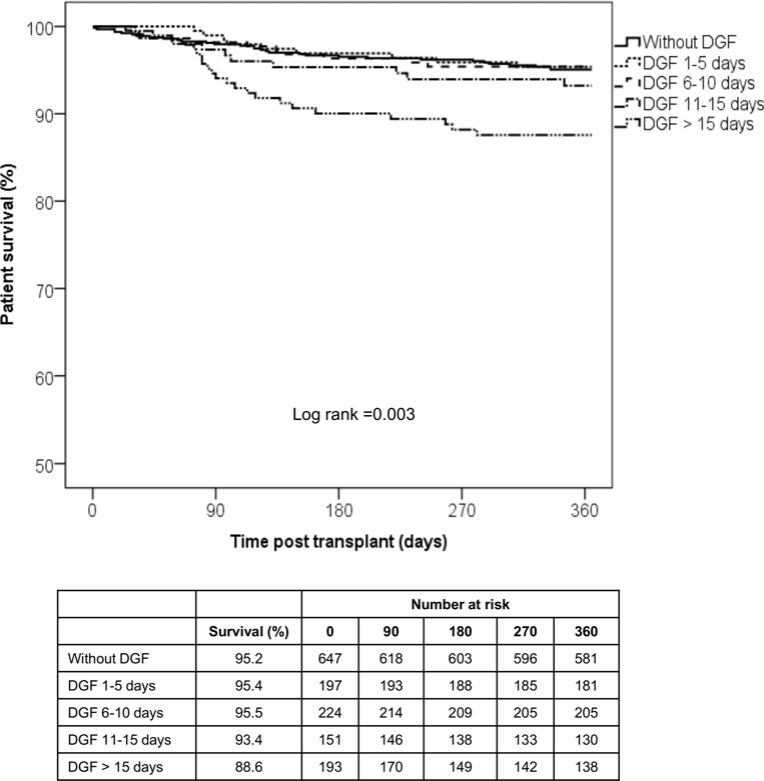
1-year patient survival according to time on DGF. Only patients with DGF longer than 15 days presented inferior patient survival.

Patients with prolonged DGF showed inferior 1-year patient survival (Fig 3) and death-censored graft survival compared to the other groups (Fig 4). Overall graft survivals were also inferior in patients with prolonged DGF (91% vs. 91.4% vs. 92% vs. 88.7% vs. 70.5%, p<0.001), respectively. There were no differences regarding the causes of graft loss and death among the groups. The predominant causes of graft loss and death were IF/TA and infection, respectively. Three patients in the prolonged DGF group lost their grafts due to primary non-function (Table 3).

**Table 3:** 1-year graft loss and death causes according to time on DGF.

	Without DGF	DGF < 5 days	DGF 5–10 days	DGF 10–15 days	DGF > 15 days	P value
**Graft loss**	**N = 28**	**N = 8**	**N = 7**	**N = 9**	**N = 39**	0.468
*Technical*	9 (32.1)	2 (25)	4 (57.1)	1 (11.1)	3 (7.7)	
*Acute rejection*	9 (32.1)	2 (25)	1 (14.3)	2 (22.2)	14 (35.9)	
*IF/TAi/ni*	6 (21.4)	1 (12.5)	0 (0)	2 (22.2)	7 (17.9)	
*PNF*	0 (0)	0 (0)	0 (0)	0 (0)	3 (7.7)	
*Infection*	2 (7.1)	2 (25)	0 (0)	2 (22.2)	5 (12.8)	
*Others*	2 (7.1)	1 (12.5)	2 (28.6)	2 (22.2)	6 (15.4)	
*Unknown*	0 (0)	0 (0)	0 (0)	0 (0)	1 (2.6)	
**Death**	**N = 31**	**N = 9**	**N = 10**	**N = 9**	**N = 24**	0.960
*Infection*	18 (58.1)	7 (77.8)	7 (70)	6 (66.7)	18 (75)	
*Cardiovascular*	10 (32.3)	1 (11.1)	2 (20)	2 (22.2)	5 (20.8)	
*Malignancy*	1 (3.2)	0 (0)	0 (0)	0 (0)	0 (0)	
*Unknown*	2 (6.5)	1 (11.1)	1 (10)	1 (11.1)	1 (4.2)	

IF/TAi/ni: immunological and no immunological interstitial fibrosis / tubular atrophy; PNF: primary non function.

Multivariable analysis showed that prolonged DGF was an independent risk factor associated with graft loss, death censored graft loss, and death at 1 year. Other risk factors for graft loss at 1 year were diabetes, PRA, donor history of hypertension and AR episodes. Risk factors associated with death censored graft loss at 1 year were PRA and AR episodes. Recipient age and donor history of hypertension were associated with death (Table 4).

**Table 4:** Multivariable analysis for risk evaluation of 1-year graft loss, death censored graft loss and death.

	Graft loss	Death censored graft loss	Death
	OR (95% CI), p	OR (95% CI), p	OR (95% CI), p
**Recipient age (years)**			1.030 (1.009–1.051), 0.005
**Recipient gender—male (yes/no)**			
**Diabetes (yes/no)**	1.813 (1.015–3.206), 0.041		
**Time on dialysis (months)**			
**PRA (%)**	1.009 (1.009–1.017). 0.022	1.013 (1.003–1.024), 0.015	
**Retransplantation (yes/no)**			
**Donor age (years)**			
**Cerebrovascular death (yes/no)**			
**Final creatinine (mg/dL)**			
**Donor–Hypertension (yes/no)**	1.805 (1.154–2.825), 0.010		2.158 (1.213–3.839), 0.009
**Cardiac arrest (yes/no)**			
**ATG induction (yes/no)**			
**CNI-based regimen (yes/no)**			
**Mycophenolate (yes/no)**			
**Cold ischaemia time (hours)**			
**Acute rejection at 6 months (yes/no)**	1.647 (1.040–2.609), 0.033	3.031 (1.686–5.448), <0.001	
**Time on DGF (yes/no)**			
Without DGF	REF	REF	REF
*1–5 days*	0.810 (0.387–1.696), 0.577	0.999 (0.384–2.596), 0.998	0.705 (0.255–1.948), 0.500
*6–10 days*	0.778 (0.385–1.572), 0.484	0.440 (0.142–1.359), 0.154	1.081 (0.458–2.550), 0.860
*11–15 days*	0.891 (0.408–1.944), 0.771	0.885 (0.313–2.502), 0.817	1.049 (0.379–2.901), 0.926
*> 15 days*	3.876 (2.270–6.618), <0.001	4.103 (2.055–8.193), <0.001	3.065 (1.536–6.117), 0.001

PRA: panel reactive antibodies; ATG: anti-thymocyte globulin; DGF: delayed graft function; CNI: calcineurin inhibitor; OR: odds ratio; CI: confidence interval.

### Renal Function

**Fig 5:**
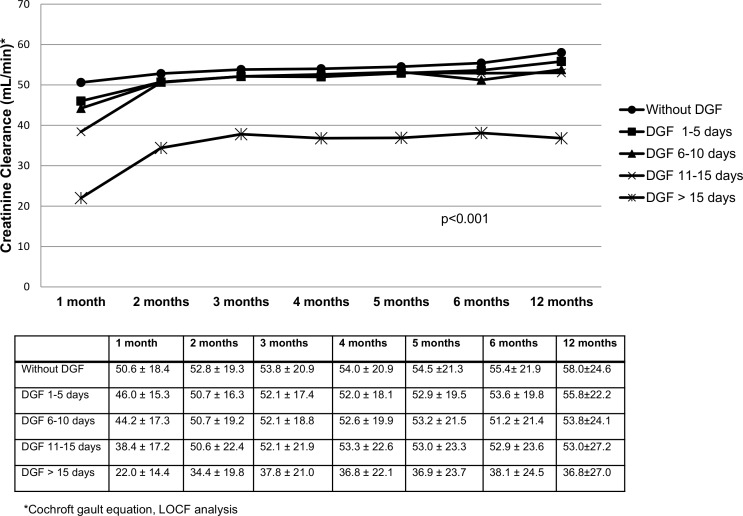
Monthly renal function according to time on DGF. Patients with prolonged DGF presented inferior renal function when compared with other groups.

Renal function at 1 month was lower in the prolonged DGF and in DGF 11–15 days groups. From 2 to 12 months no differences in renal function were detected comparing patients without DGF and those with DGF up to 15 days. Nonetheless, patients with prolonged DGF still showed inferior renal function up to one year of follow up (Fig 5).

## Discussion

In this single-center retrospective study, including a population with high incidence of DGF, we showed that patients who required dialyses for more than 15 days presented inferior clinical outcomes compared with patients with immediate renal function or shorter periods of dialysis. Importantly, these results could be observed as early as at 1 year after transplantation.

Despite the fact that our population was composed of patients with low risk of DGF, our overall incidence is at least two times higher compared with Organ Procurement and Transplantation Network (OPTN) data [13], similar to that observed in other Brazilian centers [14]. This is probably due to several factors, including delayed notification of potential donors and inadequate management of the donors before transplantation [15,16]. Importantly, this high incidence probably strengthened the study and increased its power.

Although a detrimental effect of DGF *per se* in the graft survival is probable, some authors suggest that in the absence of acute rejection, DGF had no impact on transplant outcomes [17]. In fact, in our cohort acute rejection was associated with inferior outcomes in patients with DGF. Interestingly, patients who had DGF without AR episodes showed a 7.8% superior death censored graft survival compared to patients who did not develop DGF but presented AR episodes.

As expected, the level of renal function at 1 month was inversely associated with time on DGF. Interestingly, patients without DGF and those with DGF up to 15 days recovered renal function at 2 months, reaching mean creatinine clearances above 50 mL/min but those patients with DGF longer than 15 days reached the second month after transplant with a mean creatinine clearance below 40 mL/min. From there, parallel trends were observed in all groups up to one year. Similar results were previously demonstrated in a small single center study where patients with DGF lasting more than 4 weeks presented inferior renal function compared to patients with immediate function or to patients with DGF duration inferior to 4 weeks [9].

In our cohort, only DGF longer than 15 days was associated with graft loss. Prior studies that evaluated the impact of the duration of DGF on graft survival have shown conflicting results. Yokoyama et al. observed a higher incidence of graft failure at 5 years in patients who presented DGF longer than 8 days compared to those without DGF or with DGF up to 8 days [7]. Similarly, Fernández-Juarez et al. found inferior graft survival rates at 1, 3, and 6 years in patients with DGF longer than 14 days compared to those without DGF or with DGF up to 14 days. However, when never-functioning grafts were censored or when they recalculated the graft survival rates considering only those grafts functioning at 1 year, there were no differences in graft survival among groups [8]. Moreover, analyzing recipients of non-heart beating donor kidney allografts, no impact of the duration of DGF on 5 year graft and patient survivals were observed [9]. Importantly, the effect of DGF on graft survival of heart beating donors and non-heart beating donor allografts may be completely different, and despite this population is known to be more susceptible to DGF, they present better long-term prognosis when compared to brain dead donor kidney transplant recipients [18].

Prolonged DGF was also associated with inferior patient survival in our cohort. There is no consensus on the impact of DGF or time on DGF on patient outcome. Renkens et al. found no difference in 5 year patient survival between patients with DGF longer than 2 weeks compared to patients without DGF or with DGF up to 2 weeks [9]. On the contrary, Perez Fontán et al. found that DGF longer than 3 weeks was associated with decreased patient survival, mainly due to a higher infectious mortality rate [10]. Despite the conflicting results of these studies, altogether these data suggest that the severity of the acute kidney injury is directly associated with duration of DGF, which is associated with inferior renal function, and graft and patient survivals. Multivariable analysis showed that retransplantation and HLA mismatches were independent risk factors for prolonged DGF. Interestingly, these immunological variables outperformed the favorable donor characteristics and shorter cold ischemia time observed in patients with prolonged DGF group. Prior history of transplantation is associated with sensitization, leading to increased activity of preformed anti-HLA antibodies, a known risk factor for DGF [19, 20], although in our cohort PRA was not associated with increased risk of prolonged DGF.

There are several limitations associated with this study, including its retrospective nature, wide enrollment period, peculiar donor maintenance performance, and lack of donor specific anti-HLA antibodies, precluding generalization and extrapolation of our findings.

In conclusion, in this population with high incidence of DGF, patients who develop DGF longer than 15 days presented inferior renal function, patient and graft survival at 1 year compared to patients without DGF and to patients with DGF duration shorter than 15 days. Immunological risk factors were associated with prolonged duration of DGF.

## References

[1] Yarlagadda SG, Coca SG, Formica RN, Poggio ED, Parikh CR. Association between delayed graft function and allograft and patient survival: a systematic review and meta-analysis. Nephrol Dial Transplant. 2009;24(3):1039–47. 10.1093/ndt/gfn667 19103734

[2] Butala NM, Reese PP, Doshi MD, Parikh CR. Is Delayed Graft Function Causally Associated With Long-Term Outcomes After Kidney Transplantation? Instrumental Variable Analysis. Transplantation. 2013;95(8):1008–14. 10.1097/TP.0b013e3182855544 23591726 PMC3629374

[3] Shoskes DA, Parfrey NA, Halloran PF. Increased major histocompatibility complex antigen expression in unilateral ischemic acute tubular necrosis in the mouse. Transplantation. 1990;49(1):201–7. 2105546 10.1097/00007890-199001000-00045

[4] Qureshi F, Rabb H, Kasiske BL. Silent acute rejection during prolonged delayed graft function reduces kidney allograft survival. Transplantation. 2002;74(10):1400–4. 12451239 10.1097/00007890-200211270-00010

[5] Chawla LS, Eggers PW, Star RA, Kimmel PL. Acute kidney injury and chronic kidney disease as interconnected syndromes. N Engl J Med. 2014;371(1):58–66. 10.1056/NEJMra1214243 24988558 PMC9720902

[6] Johnston O, O'kelly P, Spencer S, Donohoe J, Walshe JJ, Little DM, et al Reduced graft function (with or without dialysis) vs immediate graft function—a comparison of long-term renal allograft survival. Nephrol Dial Transplant. 2006;21(8):2270–4. 16720598 10.1093/ndt/gfl103

[7] Yokoyama I, Uchida K, Kobayashi T, Tominaga Y, Orihara A, Takagi H. Effect of prolonged delayed graft function on long-term graft outcome in cadaveric kidney transplantation. Clin Transplant. 1994;8(2 Pt 1):101–6. 8019017

[8] Fernández-Juarez G, Marcén R, Pascual J, Teruel JL, Rivera ME, Villafruela JJ, et al Prolonged delayed graft function decreases graft survival in transplant patients taking cyclosporine. Transplant Proc. 2002;34(1):338–9. 11959314 10.1016/s0041-1345(01)02789-0

[9] Renkens JJ, Rouflart MM, Christiaans MH, van den Berg-Loonen EM, van Hooff JP, van Heurn LW. Outcome of nonheart-beating donor kidneys with prolonged delayed graft function after transplantation. Am J Transplant. 2005;5(11):2704–9. 16212630 10.1111/j.1600-6143.2005.01072.x

[10] Pérez Fontán M, Rodríquez-Carmona A, Bouza P, García Falcón T, Moncalián J, Oliver J, et al Outcome of grafts with long-lasting delayed function after renal transplantation. Transplantation. 1996;62(1):42–7. 8693542 10.1097/00007890-199607150-00009

[11] Klein R, Galante NZ, de Sandes-Freitas TV, de Franco MF, Tedesco-Silva H, Medina-Pestana JO. Transplantation with kidneys retrieved from deceased donors with acute renal failure. Transplantation. 2013;95(4):611–6. 10.1097/TP.0b013e318279153c 23274968

[12] Mallon DH, Summers DM, Bradley JA, Pettigrew GJ. Defining delayed graft function after renal transplantation: simplest is best. Transplantation. 2013;96(10):885–9. 10.1097/TP.0b013e3182a19348 24056620

[13] Organ Procurement and Transplantation Network (OPTN), Scientific Registry of Transplant Recipients (SRTR). OPTN/SRTR 2012 Annual Data Report Rockville, MD: Department of Health and Human Services, Health Resources and Services Administration; 2014.

[14] Azevedo LS, Castro MC, Monteiro de Carvalho DB, d'Avila DO, Contieri F, Gonçalves RT, et al Incidence of delayed graft function in cadaveric kidney transplants in Brazil: a multicenter analysis. Transplant Proc. 2005;37(6):2746–7. 16182798 10.1016/j.transproceed.2005.05.005

[15] Baptista AP, Silva HT, Pestana JO. Influence of deceased donor hemodynamic factors in transplant recipients renal function. J Bras Nefrol. 2013;35(4):289–98. 10.5935/0101-2800.20130048 24402109

[16] Malinoski DJ, Patel MS, Ahmed O, Daly MC, Mooney S, Graybill CO, et al The impact of meeting donor management goals on the development of delayed graft function in kidney transplant recipients. Am J Transplant. 2013;13(4):993–1000. 10.1111/ajt.12090 23406284

[17] Troppmann C, Gillingham KJ, Benedetti E, Almond PS, Gruessner RW, Najarian JS, et al Delayed graft function, acute rejection, and outcome after cadaver renal transplantation. The multivariate analysis. Transplantation. 1995;59(7):962–8. 7709456 10.1097/00007890-199504150-00007

[18] Brook NR, White SA, Waller JR, Veitch PS, Nicholson ML. Non-heart beating donor kidneys with delayed graft function have superior graft survival compared with conventional heart-beating donor kidneys that develop delayed graft function. Am J Transplant. 2003;3(5):614–8. 12752318 10.1034/j.1600-6143.2003.00113.x

[19] Arias M. Impact of the delayed graft function in hypersensitized kidney transplant patients. Transplant Proc. 2003;35(5):1655–7. 12962745 10.1016/s0041-1345(03)00564-5

[20] Salamzadeh J, Sahraee Z, Nafar M, Parvin M. Delayed graft function (DGF) after living donor kidney transplantation: a study of possible explanatory factors. Ann Transplant. 2012;17(3):69–76. 23018258 10.12659/aot.883460

